# The difference of burden of ectopic beats in different types of atrial fibrillation and the effect of atrial fibrillation type on stroke risk in a prospective cohort of patients with atrial fibrillation (CODE-AF registry)

**DOI:** 10.1038/s41598-020-63370-4

**Published:** 2020-04-14

**Authors:** Seunghoon Cho, Jun Kim, Jin-Bae Kim, Junbeom Park, Jin-Kyu Park, Ki-Woon Kang, Jaemin Shim, Eue-Keun Choi, Young Soo Lee, Hyung Wook Park, Boyoung Joung

**Affiliations:** 1 Division of Cardiology, Department of Internal Medicine, Severance Cardiovascular Hospital, Yonsei University College of Medicine, Seoul, Republic of Korea; 2 Heart Institute, Asan Medical Center, University of Ulsan College of Medicine, Seoul, Republic of Korea; 3 Division of Cardiology, Department of Internal Medicine, Kyung Hee University Hospital, Kyung Hee University, Seoul, Republic of Korea; 4 Department of Cardiology, School of Medicine, Ewha Womans University, Seoul, Republic of Korea; 5 Department of Cardiology, Hanyang University Seoul Hospital, Seoul, Republic of Korea; 6 Division of Cardiology, Eulji University Hospital, Daejeon, Republic of Korea; 7 Division of Cardiology, Department of Internal Medicine, Korea University Medical Center, Seoul, Republic of Korea; 8 Department of Internal Medicine, Seoul National University Hospital, Seoul, Republic of Korea; 9 Division of Cardiology, Department of Internal Medicine, Daegu Catholic University Medical Center, Daegu, Republic of Korea; 10 Division of Cardiology, Department of Internal Medicine, Chonnam National University Hospital, Chonnam National University School of Medicine, Gwangju, Republic of Korea

**Keywords:** Cardiovascular diseases, Cardiology, Medical research

## Abstract

The relationship between atrial fibrillation (AF) type and stroke risk is still controversial. We investigated the difference of burden of atrial ectopic beats in different types of AF and the effect of the AF type on stroke risk in patients with non-valvular AF. In the prospective, multicenter observational registry with more than about 10,000 AF patients, 8883 non-valvular AF patients (mean age, 67.0 years; 36% were women) with eligible follow-up visits participated. We compared the burden of ectopic beats and stroke risk between patients with paroxysmal AF (n = 5,808) and non-paroxysmal AF (n = 3,075). The patients with a non-paroxysmal type of AF were older, male-predominant and had a higher prevalence of comorbidities and had more anticoagulation and rhythm control treatment than those with paroxysmal AF. In terms of the difference in burden of ectopic beats, patients with non-paroxysmal AF had a higher proportion of atrial premature beats (APBs) (paroxysmal vs. non-paroxysmal, median 3% vs. 5%; p = 0.001) in 24 hours Holter monitoring. During a median follow-up period of 16.8 months (Interquartile range [IQR], 11.67–20.52), a total of 82 (0.92%) patients experienced ischemic stroke with incidence rates of 0.50 and 1.09 events per 100 person-year for paroxysmal and non-paroxysmal AF, respectively. The cumulative incidence of stroke events was significantly higher in non-paroxysmal AF than in paroxysmal AF (p < 0.001). The risk of ischemic stroke was higher in non-paroxysmal AF with an adjusted hazard ratio (HR) of 2.08 (95% confidence interval [CI], 1.33–3.25; p = 0.001) than in paroxysmal AF. The type of AF was associated with an increased risk of stroke, along with the difference of burden of ectopic beats (specially in APBs) in different types of AF. These results suggest that the type of AF should be considered in stroke prevention and decision-making for oral anticoagulation in AF patients.

## Introduction

Atrial fibrillation (AF) is the most common cardiac arrhythmia, and it affects ~1–2% of the general population; its prevalence is also increasing constantly with the increasing aging population^1–4^. AF is associated with increased risk of mortality and many adverse outcomes such as stroke, thromboembolism, heart failure^5^. AF is also related to a five-fold increase in stroke risk, and one in five cases of stroke is attributed to AF^6–8^. With the increasing epidemiological burden, AF is becoming an increasingly important factor in stroke occurrence^2,3,6–8^.

To date, several studies have reported that patients with paroxysmal AF have a risk of stroke events similar to that in patients with persistent and permanent AF^9–12^. Based on the results of previous studies, the current guidelines do not include the type of AF as a risk stratification factor^13,14^. Similarly, the Korean guidelines do not include the type of AF as a risk factor. However, the association between the type of AF and stroke risk still remains controversial^15^. Several studies have shown that patients with paroxysmal AF have a lower incidence rate of stroke than those with non-paroxysmal forms of AF, although they are less likely to receive oral anticoagulant (OAC) therapy in actual clinical practice^12,16,17^. In addition to several studies dealing with the relationship between AF type and stroke risk, some studies on the relationship between implantable cardiac device-detected AF burden and thromboembolic risk, including stroke, have revealed that AF burden is associated with elevated risk for stroke and thromboembolism^18–20^. On the other hand, there have been several works on the association between the burden or frequency of atrial ectopic beats and recurrent stroke, transient ischemic attack, and death^21–24^. Results from EMBRACE Trial and several studies also have revealed that excessive APBs is associated with the development of atrial fibrillation and adverse cardiovascular events^25–27^.

This study was performed to investigate the effect of the AF type on stroke risk in patients with non-valvular AF and to investigate the difference of burden of ectopic beats in different types of AF.

## Materials and Methods

### Study design and methods

The COmparison study of Drugs for symptom control and complication prEvention of Atrial Fibrillation (CODE-AF) was a prospective, multicenter, observational study performed in patients with AF aged >18 years attending any of the 18 tertiary centers encompassing all geographical regions of Korea. The study enrollment period started in June 2016 and ended in May 2019.

The aim of the CODE-AF registry is to describe the clinical epidemiology of patients with AF and to determine the diagnostic and therapeutic processes (including organization of programs for AF management) applied to these patients and their clinical outcomes^17^.

Data collection was usually conducted by personnel with no clinical activity assigned to the project^17,28^. The Congestive heart failure/left ventricular dysfunction, Hypertension, Age of 75 years (doubled), Diabetes, Stroke (doubled), Vascular disease, 65–74 years of Age, and Sex category (female) (CHA_2_DS_2_-VASc) score and Hypertension, Abnormal renal/liver function, Stroke, Bleeding history or predisposition, Labile INR, Elderly, Drugs/alcohol concomitantly (HAS-BLED) score were calculated for all patients with non-valvular AF^17,28^. Follow-up was scheduled at every 6 months, either via personal interview or telephone contact (data not shown)^17,28^.

The registry was funded by the National Evidence-Based Healthcare Collaborating Agency and designed and coordinated by the Korea Heart Rhythm Society, which provides support to related committees, national coordinators, and participating centers. Data are entered into a common electronic database that limits inconsistencies and errors and provides online help for key variables. Each center can see its own data and data from all other participating centers^17,28^. The study was approved by the ethics committee of each center, and all patients provided informed consent for their inclusion. The study was conducted in compliance with the ethical rules of the Declaration of Helsinki as a statement of ethical principles for medical research involving human subjects by The World Medical Association. We followed the ethical, scientific and medical standards that protect the rights of participants and we required informed consent from all study participants, and the review and approval of study protocols, including patient information forms, by ethics committees.

This study was registered at ClinicalTrials.gov (NCT02786095). The Ethics Committees of all 18 tertiary centers include followings; Severance Hospital, Seoul National University Hospital, Korea University Medical Center, Daegu Catholic University Medical Center, Ewha Womans University Medical Center, Daejeon Eulji University Hospital, Kyung Hee University Hospital, Hanyang University Seoul Hospital, Chonnam National University Hospital, Asan Medical Center, Inha University Hospital, Gangnam Severance Hospital, Samsung Medical Center, CHA Bundang Medical Center, Seoul St. Mary’s Hospital, Seoul National University Bundang Hospital, Keimyung University Dongsan Medical Center, Dong-a University Hospital.

**Figure 1:**
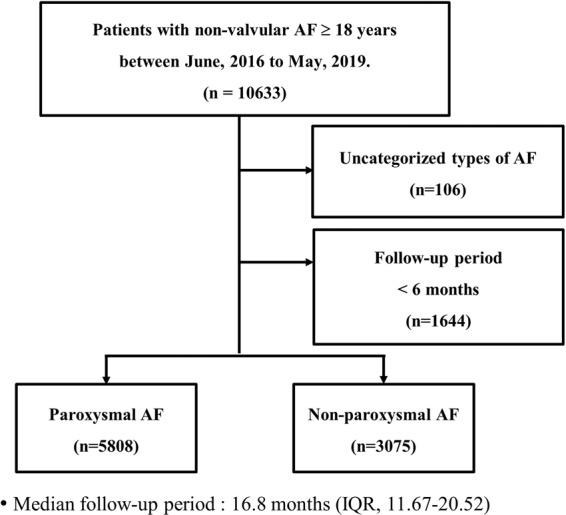
Flow diagram of the study participants. n refers to the number of patients. AF, atrial fibrillation; IQR, interquartile range.

A total of 10,663 patients older than 18 years with non-valvular AF were enrolled in the CODE-AF registry from June 2016 to May 2019. Patients with uncategorized types of AF (n = 106) and those without eligible follow-up periods (at least 6 months, n = 1,644) were excluded. Finally, 8,883 patients were enrolled in this study; the enrolled patients were categorized into two groups according to their type of AF as follows: paroxysmal AF (n = 5,808), non-paroxysmal AF (n = 3,075) (Fig. 1).

### Definition and classification of AF type

The current classification of the AF type is based on the clinical presentation and the duration and frequency of AF episodes detected by electrocardiogram (ECG) recordings at variable duration and intensity of monitoring^5^. Based on the clinical presentations of AF and taking into account the available data on arrhythmia duration, we classified and defined the type of AF as follows independently of their symptoms^5,13^.

• Paroxysmal AF, which is a self-terminating form of arrhythmia lasting less than 7 days;

• Persistent AF, which is a form of AF lasting longer than 7 days or requiring termination by cardioversion (pharmacological or electrical cardioversion) for sinus rhythm restoration;

• Permanent AF, which is a form of AF for which cardioversion is not attempted, as the arrhythmia is accepted by the patient and physicians

The patients were classified into three groups (three clinical subtypes of AF at the time of enrollment) based on their diagnosis, baseline ECG recordings, and clinical history. When comparing the stroke risks among the three groups, the total patient number was relatively very small in permanent AF (paroxysmal n = 5808, persistent n = 2806, permanent n = 269) and the baseline characteristics in permanent AF were similar to persistent AF (Supplementary Table 1). Furthermore, most of the distinction between persistent AF and permanent AF were decided subjectively by clinicians. for this reason, we combined these 2 subtypes as non-paroxysmal AF in this study to facilitate the comparative analysis according to AF type and compared baseline clinical characteristics and outcomes between paroxysmal AF and non-paroxysmal AF.

Continuous monitoring of cardiac rhythm by a Holter monitor made it possible to obtain data on the presence and duration of arrhythmias and its distribution. The burden of ectopic beats referred to this study included the percentage of atrial premature beats (APBs) and ventricular premature beats (VPBs) assessed via a Holter monitoring. We analyzed these parameters obtained by a Holter monitoring as indicators that can reflect the burden of ectopic beats.

### Study endpoint

The primary endpoint of this study was the occurrence of stroke events during the follow-up period. The other clinical endpoints included systemic thromboembolism (STE), all-cause death, and composite endpoints that contained stroke, STE, all-cause death during the follow-up period. Patients who experienced >1 stroke recurrence during the follow-up period were censored at the time of the first event. Stroke was defined as the sudden onset of a focal neurological deficit in a location consistent with the territory of the major cerebral artery, and the diagnosis of ischemic stroke was confirmed by computed tomography or magnetic resonance imaging^12^. STE was defined as an acute vascular occlusion of an extremity or major organ with related symptoms confirmed by imaging modalities such as computed tomography or ultrasonography^12^.

### Statistical analysis

Continuous variables were presented as mean ± standard deviations (SD) for normal distribution value or median [interquartile range (IQR)] for non-normal distribution value and categorical variables as numbers and percentages in each group. The baseline characteristics of the two groups were compared using student’s T-test or Mann Whitney U-test for continuous variables and Pearson’s χ^2^ test or Fisher’s exact test for categorical variables. The Kaplan–Meier method was used to estimate the cumulative incidence of stroke events and other clinical events. Differences in the cumulative incidence curves between the two groups were evaluated using the log-rank test. We used Cox regression model for multivariate analysis to assess the independent relationship between the AF type and stroke events, other clinical events adjusting for clinical variables, including age, sex, hypertension, diabetes, dyslipidemia, prior stroke/transient ischemic attack, congestive heart failure, prior myocardial infarction or peripheral arterial occlusive disease and anticoagulant, antiplatelet, statin use. Cox regression analysis was also used to confirm the statistical significance between burden of ectopic beats and stroke risk. Associations were presented as hazard ratios (HR) with 95% confidence intervals (CI). Statistical analyses were performed using the R statistical software (version 3.6.1). All reported p-values were based on two-sided tests, and p-values of <0.05 were considered statistically significant.

## Results

### Baseline characteristics

The mean age of the study population was 67 ± 11 years. Male sex was more predominant (n = 5,689, 64.0%). The median CHA_2_DS_2_-VASc score and HAS-BLED score were 3 (IQR, 1–4) and 2 (IQR, 1–2) points, respectively. The median follow-up period of the enrolled patients was 16.8 months (IQR, 11.67–20.52).

The comparison of the baseline characteristics between the two study groups is presented in Table 1. The patients with non-paroxysmal AF were older, male-predominant and had a higher prevalence of comorbidities such as hypertension, diabetes, history of stroke and transient ischemic attack, and valvular heart disease and congestive heart failure and also had higher CHA_2_DS_2_-VASc score and larger left atrium size (all p < 0.05) than the patients with paroxysmal AF. Patients in the non-paroxysmal AF group were also more frequently treated with OAC therapy and rhythm control medications than those in the paroxysmal AF group. On the other hand, patients with paroxysmal AF were more prescribed with aspirin and had more history of catheter ablation.

**Table 1:** Baseline characteristics according to each type of AF.

Clinical variable	Paroxysmal AF (n = 5808)	Non-paroxysmal AF (n = 3075)	p-value
Age in years	66.55 ± 11.03	68.00 ± 10.73	<0.001
Female sex (%)	2160 (37.2)	1034 (33.6)	0.001
Body mass index (kg/m^2^)	24.51 ± 3.28	24.94 ± 3.46	<0.001
Systolic BP (mmHg)	122.43 ± 15.10	122.59 ± 15.76	0.637
Diastolic BP (mmHg)	74.66 ± 11.44	75.64 ± 12.21	<0.001
Heart rate (/min)	74.18 ± 16.54	79.61 ± 16.89	<0.001
Hypertension (%)	3804 (65.6)	2124 (69.2)	0.001
Diabetes (%)	1399 (24.1)	839 (27.4)	0.001
Dyslipidemia (%)	2148 (37.0)	996 (32.6)	<0.001
History of stroke/TIA (%)	837 (14.4)	509 (16.6)	0.008
History of myocardial infarction (%)	182 (3.1)	77 (2.5)	0.108
Valvular heart disease (%)	409 (7.1)	419 (13.7)	<0.001
Congestive heart failure (%)	411 (7.1)	470 (15.3)	<0.001
Chronic kidney disease (%)	576 (9.9)	300 (9.8)	0.84
End-stage renal disease (%)	101 (1.8)	40 (1.3)	0.132
Peripheral arterial occlusive disease (%)	320 (5.5)	178 (5.8)	0.622
Cancer (%)	528 (9.1)	358 (11.6)	<0.001
CHA_2_DS_2_-VASc score	2 [1–4]	3 [2–4]	<0.001
HAS-BLED score	2 [1–2]	2 [1–2]	0.182
NT-proBNP (ng/mL)	0.44 [0.13–1.72]	0.93 [0.34–2.59]	<0.001
Troponin T (ng/mL)	0.01 [0.01–0.02]	0.01 [0.01–0.02]	0.002
LA size (mm)	41.0 [37.0–46.8]	46.0 [42.0–51.4]	<0.001
LAVI (mL/m^2^)	37.1 [28.3–51.9]	50.0 [38.8–64.0]	<0.001
LV ejection fraction (%)	63.0 [58.0–68.0]	60.0 [55.0–65.0]	<0.001
E/E’	10.1 [8.0–13.3]	11.0 [8.4–14.2]	<0.001
Implantable cardiac device (%)	420 (7.2)	117 (3.8)	<0.001
History of catheter ablation (%)	933 (16.1)	368 (12.0)	<0.001
History of cardioversion (%)	672 (11.6)	690 (22.5)	<0.001
**Medications**			
Warfarin (%)	950 (16.4)	641 (20.8)	<0.001
NOAC (%)	1753 (30.2)	1177 (38.3)	<0.001
Aspirin (%)	1060 (18.3)	470 (15.3)	<0.001
Clopidogrel (%)	377 (6.5)	196 (6.4)	0.866
Cilostazol (%)	28 (0.5)	14 (0.5)	0.99
Beta-blocker (%)	2691 (46.3)	1639 (53.3)	<0.001
Calcium channel blocker (%)	1582 (27.2)	902 (29.3)	0.039
Statin (%)	1991 (34.3)	1043 (33.9)	0.75
ARB/ACEi (%)	2124 (36.6)	1413 (46.0)	<0.001
Class Ic AAD (%)	2282 (39.3)	723 (23.5)	<0.001
Class III AAD (%)	790 (13.6)	494 (16.1)	0.002
Digitalis (%)	313 (5.4)	292 (9.5)	<0.001

Categorical data are presented as numbers (%) and continuous data are presented as mean ± standard deviations for normal distribution value or median [interquartile range] for non-normal distribution value.

AAD, anti-arrhythmic drug; ACEi, angiotensin converting enzyme inhibitor; AF, atrial fibrillation; ARB, angiotensin receptor blocker; BP, blood pressure; E/E’, early diastolic transmitral velocity (E) to early myocardial velocity (E’) ratio; LA, left atrium; LAVI, left ventricular volume index; LV, left ventricle; NOAC, non-vitamin K oral anticoagulant; NT-proBNP, N-terminal pro B-type natriuretic peptide; TIA, transient ischemic attack.

### Differences of burden of ectopic beats in different types of AF

A total of 4,861 patients without permanent AF underwent a Holter monitoring. The burden of ectopic beats was obtained through a Holter monitor and it was assessed by proportion and average of APBs and VPBs. We used these results to compare the ectopic burden between the two study groups and to assess the relationship between the ectopic burden and risk of clinical events including stroke.

**Figure 2:**
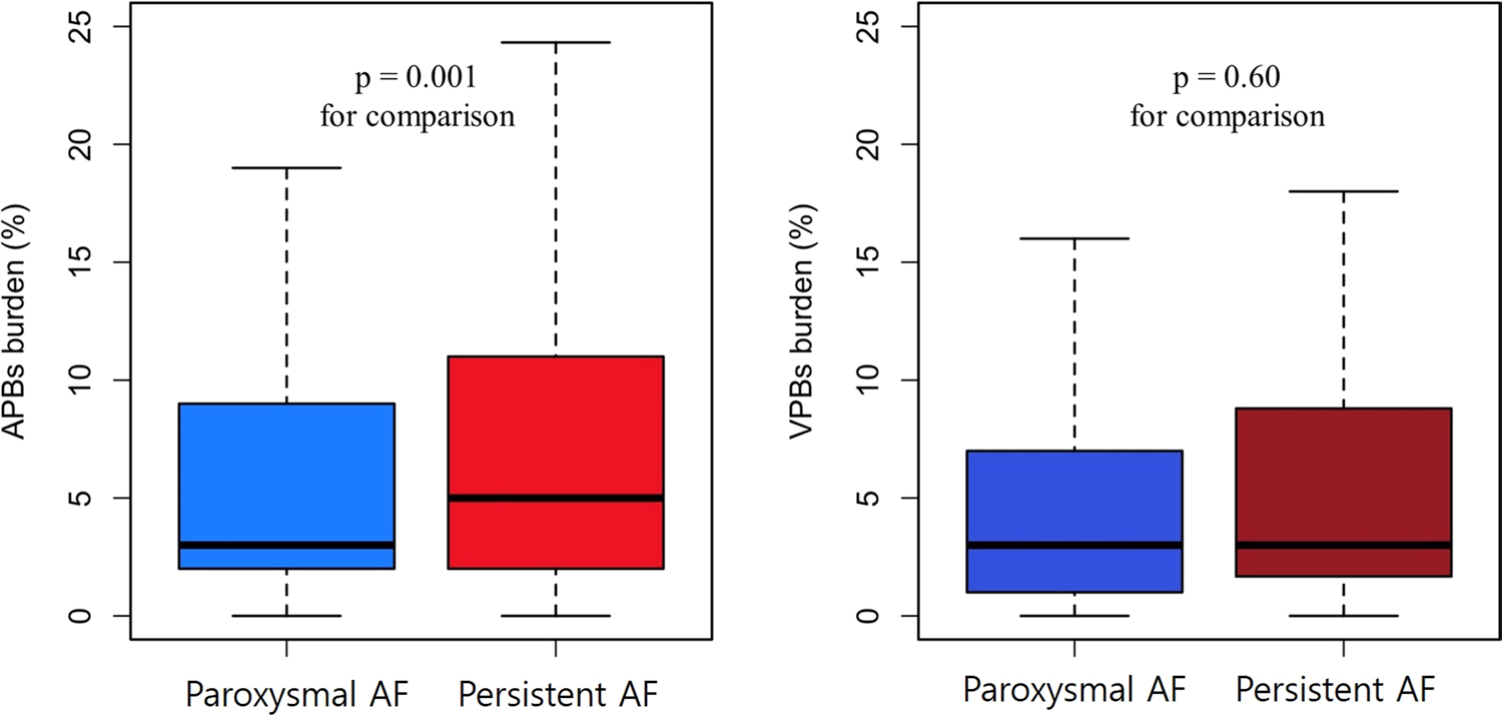
Differences in burden of atrial and ventricular ectopic beats according to types of AF on 24 hours Holter monitoring. Box plots represent the median percentage of ectopic beats (%) and interquartile range on a Holter monitor for each type of AF. AF, atrial fibrillation; APBs, atrial premature beats; PAF, paroxysmal AF; VPBs, ventricular premature beats.

The APB burden was significantly higher in non-paroxysmal AF (median, 5%; IQR, 2–11%) than in paroxysmal AF (median, 3%; IQR, 2–9%). The VPBs burden in non-paroxysmal AF was similar (median, 3%; IQR, 1.7–8.8%) to paroxysmal AF (median, 3%; IQR, 1–7%), and not statistically significant (Fig. 2).

### Multivariate analysis and Other outcomes

During a median follow-up period of 16.8 months (IQR, 11.67–20.52), a total of 82 (0.92%) patients experienced ischemic stroke with incidence rates of 0.50 and 1.09 events per 100 person-year for paroxysmal and non-paroxysmal AF, respectively. Incidence rates of STE events per 100 person-year were 0.07 and 0.15 for paroxysmal and non-paroxysmal AF, respectively. In all-cause death, incidence rates per 100 person-year were 0.48 and 0.68 for non-paroxysmal AF and paroxysmal AF. In the composite outcome, incidence rates per 100 person-year were 1.14 and 2.03 for non-paroxysmal AF and paroxysmal AF (Table 2). In subgroup without persistent AF, incidence and risk of stroke and other outcome were higher in persistent than paroxysmal AF (Supplementary Table 2).

**Table 2:** Incidence rates and adjusted HR of stroke and other clinical events according to AF type in Cox regression analysis.

Endpoint	Incidence rate (per 100 person-year)		HR (95% CI)		p-value
	Paroxysmal AF	Non-paroxysmal AF	unadjusted	clinical variable-adjusted**	
**Primary endpoint**					
Stroke	0.50	1.09	2.19 (1.42–3.39)*	2.08 (1.33–3.25)	0.001
**Other clinical endpoints**					
STE	0.07	0.15		1.35 (0.40–4.53)	0.627
All-cause death	0.48	0.68		1.24 (0.75–2.05)	0.399
Composite outcome (Stroke, STE, All-cause death)	1.14	2.03		1.61 (1.18–2.20)	0.002

*p < 0.001 for unadjusted HR in stroke risk.

**Adjusted for age, sex, hypertension, diabetes, dyslipidemia, prior stroke/transient ischemic attack, congestive heart failure, prior myocardial infarction/peripheral arterial occlusive disease, and anticoagulant, antiplatelet, statin use.

AF, atrial fibrillation; CI, confidence interval; HR, hazard ratio; STE, systemic thromboembolism.

**Figure 3:**
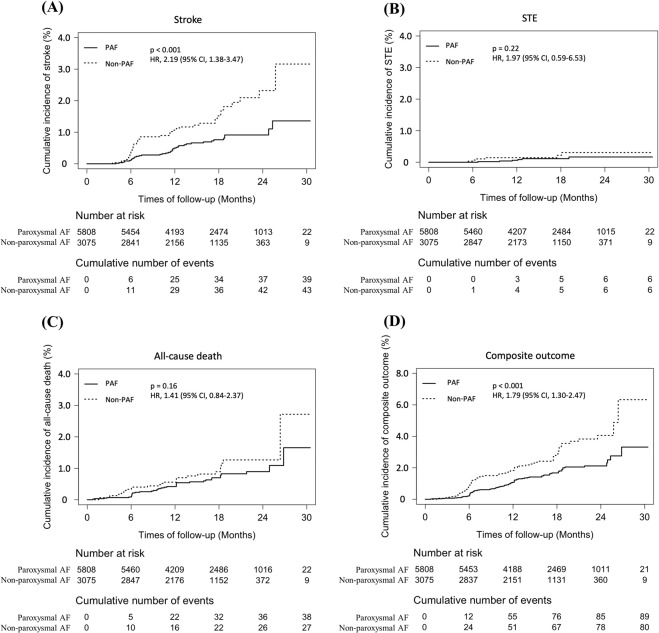
Cumulative incidence curves of primary outcome and other clinical outcomes in patients with different types of AF. (**A**) Stroke, (**B**) STE, (**C**) All-cause death, (**D**) Composite outcome. AF, atrial fibrillation; PAF, paroxysmal AF; STE, systemic thromboembolism.

The cumulative incidence of stroke was significantly higher in non-paroxysmal AF than in paroxysmal AF (p < 0.001). There was no significant difference in the cumulative incidence of STE between the two AF groups (p = 0.22). The cumulative incidence of all-cause death was slightly different as the follow-up period increased, but there was no statistical significance (p = 0.16). lastly, the cumulative incidence of composite outcomes was statistically higher in non-paroxysmal AF (p < 0.001) (Fig. 3).

In the Cox regression analysis, the patients with non-paroxysmal AF had a higher risk of ischemic stroke than the patients with paroxysmal AF during the follow-up period. The unadjusted HR was 2.19 (95% CI, 1.42–3.39; p < 0.001). After adjustment for clinical variables, the type of AF remained an independent risk factor for ischemic stroke with an adjusted HR of 2.08 (95% CI, 1.33–3.25; p = 0.001). Adjusted HRs of STE and all-cause death were 1.35 (95% CI, 0.40–4.53; p = 0.627) and 1.24 (95% CI, 0.75–2.05; p = 0.399), respectively without statistical significance. However, the risk of the composite outcome was significantly higher in non-paroxysmal AF patients with an adjusted HR of 1.61 (95% CI, 1.18–2.20; p = 0.002) (Table 2).

### Subgroup analysis for stroke risk

A subgroup analysis was performed to identify the association between the ectopic burden and stroke risk in 4,861 patients without permanent AF who underwent a Holter monitoring. Although the differences of APB burden measured by a Holter monitoring between two study groups were statistically significant, there was no significant relationship between increased APB burden, and stroke risk and other clinical event risks (Table 3).

**Table 3:** Incidence rates and adjusted HR of stroke and other clinical events according to APB burden of ectopic beats in a subgroup without permanent AF.

Endpoint	Incidence rate (per 100 person-year)		HR (95% CI)	p-value
	Low APB burden (APB < 5%)	High APB burden (5% ≤ APB)	clinical variable-adjusted**	
**Primary endpoint**				
Stroke	0.76 (0.16–0.22)	0.94 (0.19–2.74)	1.20 (0.23–6.08)	0.824
**Other clinical endpoints**				
STE	0.25 (0.01–1.41)	0	—	—
All-cause death	0.76 (0.16–2.21)	0.63 (0.76–2.26)	1.19 (0.18–7.72)	0.855
Composite outcome (Stroke, STE, All-cause death)	6.95 (3.18–13.2)	5.44 (2.0–11.9)	0.85 (0.30–2.42)	0.764

**Adjusted for age, sex, hypertension, diabetes, dyslipidemia, prior stroke/transient ischemic attack, congestive heart failure, prior myocardial infarction/peripheral arterial occlusive disease, and anticoagulant, antiplatelet, statin use.

AF, atrial fibrillation; APB, atrial premature beat; CI, confidence interval; HR, hazard ratio; STE, systemic thromboembolism.

**Figure 4:**
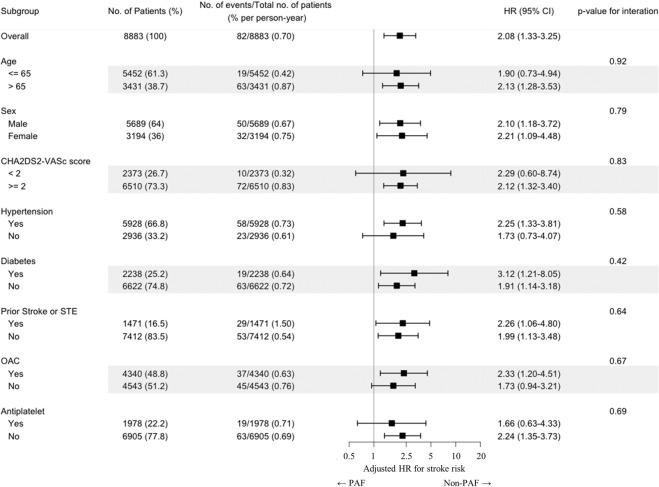
A subgroup analysis representing the adjusted HR of non-paroxysmal AF compared to paroxysmal AF in each subgroup when grouping by each clinical variable. AF, atrial fibrillation; CI, confidence interval; HR, hazard ratio; OAC, oral anticoagulants; PAF, paroxysmal AF; STE, systemic thromboembolism.

To investigate how the AF type influences stroke risk according to different subgroups, we performed subgroup analysis of stroke risk by dividing the study patients according to several clinical variables (Fig. 4). The adjusted HR for stroke represented the relative risk ratio of non-paroxysmal AF to stroke incidence compared to paroxysmal AF during the follow-up period in each patient subgroups divided by clinical variable.

In the subgroup of low CHA_2_DS_2_-VASc scores (<2, In current guidelines, not requiring anticoagulant therapy) and the subgroups with younger age (Age ≤ 65) and no anticoagulation and without hypertension, there was no statistical significance with the adjusted stroke risk of non-paroxysmal AF compared to paroxysmal AF. However, the overall adjusted HR for stroke was statistically significant, and there were no significant in-group differences in the effects of AF type on stroke occurrence in any subgroup, including age, sex, other comorbidities, and anticoagulant, antiplatelet use.

## Discussion

### Main findings

The main finding of the present study was that the patients with non-paroxysmal AF had a significantly higher risk of stroke than the patients with paroxysmal AF. Even after adjustment of confounding factors, the effect of AF type on stroke risk was still significant and the AF type still was an important factor in stroke occurrence. The Holter monitor results of 5,013 patients revealed that the APB burden was significantly different depending on the AF type, especially higher in patients with non-paroxysmal AF. However, there was no significant direct association between increasing burden of ectopic beats and stroke risk. The reason for more ectopy in the non-paroxysmal AF in this study might be explained by the factor that ectopy is more common in patients with structural and more severe heart disorders. Actually, the non-paroxysmal AF group in this study had more comorbidities.

In the multivariate regression analysis of other clinical events, the results showed that the composite outcome had a significant HR for patients with non-paroxysmal AF. Of course, this was due to a relatively high incidence of stroke in the composite outcome, but the difference in mortality during the follow-up period between the two groups was also thought to have some effect.

In subgroup analysis, there were no significant in-group differences between subgroups in the effect of AF type on stroke occurrence. These results suggest that the AF type might be related to the stroke risk independently of different age and different CHA_2_DS_2_-VASc score. So, it means that the type of AF should be considered in stroke prevention and application of OAC therapy, particularly in young AF patients with low CHA_2_DS_2_-VASc score who not requiring anticoagulant therapy in current guidelines.

### Controversy over AF type, AF burden, and stroke risk

The stroke risk in the different types of AF has been controversial. Historically, the risk of ischemic stroke has been considered to be regardless of the AF type^9,29,30^. Patients with a substantial clinical risk of stroke (CHA_2_DS_2_-VASc score, ≥2) have been anticoagulated regardless of their AF type. In contrast, several studies showed that the risk of stroke was lower in anticoagulated patients with paroxysmal AF than in those with non-paroxysmal AF^12,16,31^.

The SOS AF project, a pooled analysis of three prospective studies with 10,016 patients who were implanted with electronic devices, reported that the device-detected AF burden was associated with an increased risk of ischemic stroke after adjusting for the CHA_2_DS_2_ score and anticoagulant use^32^. In the ASSERT study, device-detected atrial tachyarrhythmias (atrial rate, >190 bpm for >6 min) were associated with an increased risk of ischemic stroke or systemic embolism during a 2.5-year follow-up period^33,34^. In the KP-RHYTHM Study, a greater burden of AF identified using a noninvasive, 14-day continuous patch devices monitoring is associated with a higher risk of ischemic stroke and thromboembolism in adults with paroxysmal AF^35^. A recent study from Kaplan *et al*. has shown that the duration of AF and CHA_2_DS_2_-VASc scores are both important components in assessing stroke risk and therefore in determining when OAC therapy should be initiated^36^.

The current practice guidelines recommend a risk-stratification approach for stroke and using OAC treatment based on the CHA_2_DS_2_-VASc score in patients with AF^13,14,29^. However, deciding whether to offer anticoagulation to patients with a lower risk of stroke is less clear. Further, the application of OAC therapy in actual clinical practice varies depending on the patients’ age, underlying disease, bleeding risk, and life expectancy^17^. For patients with a lower risk for whom the risk-benefit ratio of anticoagulation is less clear, it could be useful to consider the type of AF for decision-making regarding OAC therapy^5^.

The relationship between the AF type or burden and the stroke risk, independently of the CHA_2_DS_2_-VASc score, is still unclear^19^. Despite the heterogeneity and uncertainty of outcomes in various studies dealing with the relationship between the AF type and stroke risk, considering the current guidelines and the results from several studies including this study, the present study suggests that the current stroke risk prediction model in patients with AF, in which the risk of thromboembolic events is equivalent in different types of AF, may need to be re-evaluated; further, the type of AF should be considered as an additional risk factor to prevent ischemic stroke. Further studies are needed to evaluate the impact of the integration of the AF type into thromboembolic risk models.

### Study limitations

There are several limitations in this study. First, although the present study was a prospective study that included a large patient population and had a regular patient follow-up for every six months, the number of clinical events including stroke was rather small and the duration of follow-up was relatively short. The stroke incidence in this large population of patients was only 0.92%. The relatively low stroke rate of this study population can be explained by several factors. Compared with recent non-vitamin K oral anticoagulant (NOAC) studies, this study included patients with younger age and lower stroke risk factors (Supplementary Table 3). Our previous study showed that OAC was used in 82.7% of patients, and NOAC in more than two-third of OAC prescribed patients in this registry^17^. Moreover, persistence to OAC declined for 6 months only to 95.5% for NOAC^37^. Therefore, high OAC rate with high NOAC usage, and high persistent rate might be related with low stroke rate in this study. Second, the relationship between AF burden and stroke was not properly evaluated in this study. AF burden cannot be accurately assessed by a Holter monitor, due to the known daily variation in frequency^38^. Prolonged rhythm surveillance with implantable cardiac devices is necessary to assess the burden accurately^19,39^. We also could not find a statistically significant relationship between this burden of ectopic beats and stroke incidence. Third, in this study, the type of AF was assessed by clinician assessment. However, continuous arrhythmia monitoring might improve the accuracy of the type of AF. Clinician assessment of rhythm type is an imprecise science, particularly in the absence of continuous arrhythmia monitoring. Prior work from Charitos *et al*. shows a lack of concordance between clinical assessment and actual rhythm status as monitored by implantable devices^40^. Fourth, in baseline characteristics, there were somewhat differences in clinical variables such as age, sex, comorbidities and drug use between the two study groups. Although important factors that can affect the outcome have been adjusted, there may still be significant confounders such as the proportion of patients appropriately treated with OAC and other major comorbidities which may act as another important factor. Fifth, the antithrombotic drugs and doses were selected at the discretion of the attending physician and there was a lack of data about drug use and drug compliance during the follow-up period. And lastly, we did not consider the progression of AF during the follow-up period after determining the type of AF at the time of enrollment. A recent study showed that the risk of stroke was lower in patients with maintained paroxysmal AF than in those with paroxysmal AF at baseline that progressed to persistent or permanent AF at the 3-year follow-up^41^.

## Conclusion

This prospective, multicenter large-scale registry study revealed that the patients with non-paroxysmal AF had a significantly higher burden of ectopic beats than those with paroxysmal AF and that non-paroxysmal AF was associated with an increased risk of ischemic stroke compared with paroxysmal AF. In conclusion, the type of AF should be considered in stroke prevention and decision-making for oral anticoagulation in AF patients. Further investigations to understand the role of AF type in stroke risk will be needed. New treatment to prevent AF progression may be important to prevent stroke incidence and improve the survival of AF patients.

## Supplementary information

Supplementary Information.
