# Supplementary material for: The difference of burden of ectopic beats in different types of atrial fibrillation and the effect of atrial fibrillation type on stroke risk in a prospective cohort of patients with atrial fibrillation (CODE-AF registry)
Source: Sci Rep. 2020 Apr 14;10:6319. doi: 10.1038/s41598-020-63370-4 (PMC7156648; doi:10.1038/s41598-020-63370-4)
Supplement: Supplementary file 1 — Supplementary Information. [file 41598_2020_63370_MOESM1_ESM.docx]

***Supplement information of the manuscript***

**The difference of burden of ectopic beats in different types of atrial fibrillation and the effect of atrial fibrillation type on stroke risk in a prospective cohort of patients with atrial fibrillation (CODE-AF registry)**

Seunghoon Cho, MD^1*^; Jun Kim, MD^2*^; Jin-Bae Kim, MD^3^; Junbeom Park, MD^4^; Jin-Kyu Park, MD^5^; Ki-Woon Kang, MD^6^; Jaemin Shim, MD^7^; Eue-Keun Choi, MD^8^; Young Soo Lee, MD^9^; Hyung Wook Park, MD^10†^; and Boyoung Joung, MD^1†^

^1^Division of Cardiology, Department of Internal Medicine, Severance Cardiovascular Hospital, Yonsei University College of Medicine, Seoul, Republic of Korea

^2^Heart Institute, Asan Medical Center, University of Ulsan College of Medicine, Seoul, Republic of Korea

^3^Division of Cardiology, Department of Internal Medicine, Kyung Hee University Hospital, Kyung Hee University, Seoul, Republic of Korea

^4^Department of Cardiology, School of Medicine, Ewha Womans University, Seoul, Republic of Korea

^5^Department of Cardiology, Hanyang University Seoul Hospital, Seoul, Republic of Korea

^6^Division of Cardiology, Eulji University Hospital, Daejeon, Republic of Korea

^7^Division of Cardiology, Department of Internal Medicine, Korea University Medical Center, Seoul, Republic of Korea

^8^Department of Internal Medicine, Seoul National University Hospital, Seoul, Republic of Korea

^9^Division of Cardiology, Department of Internal Medicine, Daegu Catholic University Medical Center, Daegu, Republic of Korea

^10^Division of Cardiology, Department of Internal Medicine, Chonnam National University Hospital, Chonnam National University School of Medicine, Gwangju, Republic of Korea

^*^These authors contributed equally to this work.

^†^Joint senior authors

**Address for correspondence:**

Boyoung Joung, MD, PhD

Division of Cardiology, Department of Internal Medicine, Severance Cardiovascular Hospital, Yonsei University College of Medicine, 50-1 Yonsei-ro, Seodaemun-gu, Seoul 03722, Republic of Korea

Phone: +82-2-2228-8460, Fax: +82-2-393-2041, E-mail: cby6908@yuhs.ac

Hyung Wook Park, MD

Division of Cardiology, Department of Internal Medicine, Chonnam National University Hospital, Chonnam National University School of Medicine, 42 Jebong-ro, Dong-gu, Gwangju 61469, Republic of Korea

Tel: +82-62-220-6246, Fax: +82-62-663-3105, E-mail: mdhwp@chol.com

**Supplementary Table 1. Baseline characteristics according to three types of AF.**

|  | Paroxysmal AF  (n=5,808) | Persistent AF  (n=2,806) | Permanent AF  (n=269) | p-value |
| --- | --- | --- | --- | --- |
| Age (years) | 67 ± 11 | 68 ± 11 | 71 ± 9 | <0.001 |
| Male | 3648 (62.8) | 1861 (66.3) | 180 (66.9) | 0.004 |
| Body mass index (kg/m^2^) | 24.5 ± 3.3 | 24.9 ± 3.5 | 25.0 ± 3.4 | <0.001 |
| Systolic BP (mmHg) | 122 ± 15 | 123 ± 16 | 121 ± 15 | 0.158 |
| Diastolic BP (mmHg) | 75 ± 11 | 76 ± 12 | 75 ± 12 | <0.001 |
| Heart rate (/min) | 74 ± 21 | 79 ± 17 | 81 ± 17 | <0.001 |
| Hypertension | 3804 (65.5) | 1937 (69.0) | 187 (69.5) | 0.014 |
| Diabetes | 1399 (24.1) | 763 (27.2) | 76 (28.3) | 0.006 |
| Dyslipidemia | 2148 (37.0) | 911 (32.5) | 85 (31.6) | <0.001 |
| History of stroke/TIA | 837 (14.4) | 460 (16.4) | 49 (18.2) | 0.01 |
| History of myocardial infarction | 182 (3.1) | 66 (2.4) | 11 (4.1) | 0.179 |
| Valve disease | 409 (7.0) | 348 (12.4) | 71 (26.4) | <0.001 |
| Congestive heart failure | 411 (7.1) | 405 (14.4) | 65 (24.2) | <0.001 |
| Chronic kidney disease | 576 (9.9) | 272 (9.7) | 28 (10.4) | 0.937 |
| End stage renal disease | 101 (1.7) | 39 (1.4) | 1 (0.4) | 0.202 |
| Peripheral arterial occlusive disease | 528 (9.1) | 23 (8.6) | 886 (10.0) | 0.202 |
| Cancer | 528 (9.1) | 335 (11.9) | 23 (8.6) | <0.001 |
| CHA_2_DS_2_-VASc score | 2.6 ± 1.6 | 2.8 ± 1.7 | 3.2 ± 1.6 | <0.001 |
| HAS-BLED score | 1.8 ± 1.1 | 1.9 ± 1.1 | 2.0 ± 1.0 | 0.065 |
| NT-proBNP (ng/mL) | 87.2 ± 1251.8 | 204.1 ± 1259.4 | 100.9 ± 341.0 | 0.368 |
| Troponin T (ng/mL) | 0.08 ± 0.61 | 0.10 ± 0.50 | 0.03 ± 0.06 | 0.801 |
| LA size (mm) | 42 ± 8 | 46 ± 8 | 49 ± 7 | <0.001 |
| LAVI (mL/m^2^) | 43.3 ± 23.6 | 54.1 ± 26.5 | 62.3 ± 27.9 | <0.001 |
| LV ejection fraction (%) | 62 ± 9 | 59 ± 10 | 57 ± 10 | <0.001 |
| E/E’ | 11.7 ± 12.6 | 12.2 ± 6.4 | 12.1 ± 4.6 | 0.153 |
| Implantable cardiac device | 489 (8.4) | 124 (4.4) | 23 (8.6) | 0.001 |
| History of catheter ablation | 933 (16.1) | 345 (12.3) | 23 (8.6) | <0.001 |
| History of cardioversion | 672 (11.6) | 616 (22.0) | 74 (27.5) | <0.001 |
| Medications |  |  |  |  |
| Warfarin | 950 (16.4) | 576 (20.5) | 65 (24.2) | <0.001 |
| NOAC | 1753 (30.2) | 1079 (38.5) | 98 (36.4) | <0.001 |
| Aspirin | 1060 (18.3) | 433 (15.4) | 37 (13.8) | 0.002 |
| Clopidogrel | 377 (6.5) | 185 (6.6) | 11 (4.1) | 0.273 |
| Beta-blocker | 2691 (46.3) | 1484 (52.9) | 155 (57.6) | <0.001 |
| Calcium channel blocker | 1582 (27.2) | 809 (28.8) | 93 (34.6) | 0.015 |
| Statin | 1991 (34.3) | 944 (33.6) | 99 (36.8) | 0.547 |
| ARB/ACEi | 2300 (39.6) | 1127 (40.2) | 110 (40.9) | 0.825 |
| Class Ic AAD | 2424 (41.7) | 770 (27.4) | 22 (8.2) | <0.001 |
| Sotalol | 58 (1.0) | 29 (1.0) | 1 (0.4) | 0.575 |
| Amiodarone | 515 (8.7) | 427 (15.2) | 8 (3.0) | <0.001 |
| Dronedarone | 224 (3.9) | 37 (1.3) | 1 (0.4) | <0.001 |
| Digitalis | 313 (5.4) | 260 (9.3) | 32 (11.9) | <0.001 |

Categorical data are presented as number (%), Continuous data are presented as mean ± standard deviations.

AAD, anti-arrhythmic drugs; ACEi, angiotensin converting enzyme inhibitor; AF, atrial fibrillation; ARB, angiotensin receptor blocker; BP, blood pressure; E/E’, early diastolic transmitral velocity (E) to early myocardial velocity (E’) ratio; LA, left atrium; LAVI, left ventricle volume index; LV, left ventricle; NOAC, non-vitamin K oral anti-coagulant; NT-proBNP, N-terminal pro B-type natriuretic peptide; PYRs, pack years; TIA, transient ischemic attack.

**Supplementary Table 2. Incidence rates and adjusted HR of stroke and other clinical events of paroxysmal or persistent AF (in subgroup without permanent AF).**

|  | **Incidence rate**  **(per 100 person-year)** | | Hazard ratio  (95% CI) * | p-value |
| --- | --- | --- | --- | --- |
|  | Paroxysmal AF | Persistent AF |  |  |
| **Primary endpoint** |  |  |  |  |
| Stroke | 0.56 (0.41-0.76) | 1.39 (1.02-1.84) | 1.88 (1.18-3.0) | 0.008 |
| **Other clinical endpoints** |  |  |  |  |
| STE | 0.08 (0.03-0.18) | 0.14 (0.05-0.34) | 1.18 (0.33-4.28) | 0.801 |
| All-cause death | 0.48 (0.34-0.67) | 0.75 (0.49-1.10) | 1.18 (0.71-1.97) | 0.53 |
| Composite outcome  (Stroke, STE, All-cause death) | 3.89 (3.13-4.78) | 5.91 (4.63-7.43) | 1.49 (1.08-2.05) | 0.016 |

* Adjusted for age, sex, hypertension, diabetes, dyslipidemia, prior stroke/transient ischemic attack, congestive heart failure, prior myocardial infarction/peripheral arterial occlusive disease, and anticoagulant, antiplatelet, statin use.

AF, atrial fibrillation; CI, confidence interval; HR, hazard ratio; STE, systemic thromboembolism.

**Supplementary Table 3. Comparison of the incidence rate of stroke, age, and prevalence of stroke risk factors with NOAC randomized-controlled trials.**

|  | **CODE-AF** | **RELY** | **ROCKET** | **ARISTOTLE** | **ENGAGE** |
| --- | --- | --- | --- | --- | --- |
| Incidence of stroke | 1.38% in warfarin group  0.65% in NOAC group | 1.11% per year in 150 mg dabigatran group | 1.7% per year in rivaroxaban group | 1.27% per year in the apixaban group | 1.18% with high-dose edoxaban group |
| Age | 67 0 ± 10.9 | 71.5 ± 8.8 | 73 [65-78] | 70 [63-76] | 72 [64-78] |
| CHADS_2_ | 1.9 ± 1.2 | 2.2 ± 1.2 | 3.48 ± 0.94 | 2.1 ± 1.2 | 2.8 ± 1.0 |
| CHA_2_DS_2_-VASc score | 2 [1-4] |  |  |  |  |

Categorical data are presented as number (%), Continuous data are presented as mean ± standard deviations or median [interquartile range].

NOAC, non-vitamin K oral anti-coagulant.
